# Determinants of and socio-economic disparities in self-rated health in China

**DOI:** 10.1186/s12939-016-0496-4

**Published:** 2017-01-11

**Authors:** Jiaoli Cai, Peter C. Coyte, Hongzhong Zhao

**Affiliations:** 1 School of Economics, Wuhan University of Technology, 122 Luoshi Road, Wuhan, Hubei Province 430070 People’s Republic of China; 2 Institute of Health Policy, Management and Evaluation, University of Toronto, Health Sciences Building,155 College Street, Suite 425, Toronto, ON M5T 3M6 Canada

**Keywords:** China, Self-rated health, Socio-economic status, Lifestyle, Psychosocial factors

## Abstract

**Background:**

Self-rated health (SRH) is not only used to measure health status and health inequalities, but also as a strong predictor of morbidity and mortality. The purpose of this study was to: 1) evaluate the factors that account for variations in self-rated health among Chinese citizens; and to 2) explore the process through which socio-economic status may impact self-rated health.

**Methods:**

Data were derived from the Chinese General Social Survey (CGSS) (2013). Determinants of self-rated health were analyzed along four main dimensions: demographic characteristics, socio-economic status, lifestyle, and psychosocial factors. Multivariate odds ratios for good self-rated health were calculated for different variables in order to analyze the determinants. Binary logistic regression analysis was performed to assess the extent to which lifestyle and psychosocial factors explained the association between socio-economic status and self-rated health.

**Results:**

About 65% of the survey respondents reported good self-rated health. Women, the elderly, married or single respondents and residents of Western China were less likely to report good self-rated health. Respondents who were engaged in work, had higher household income, reported high social class and higher socio-economic status compared with peers were more likely to report good self-rated health. Normal weight and physically active respondents along with those reporting a happy life, no depression, and good relationships with families and friends were related to good self-rated health. We also found the effect of socio-economic status on self-rated health was partly explained by lifestyle and psychosocial factors.

**Conclusion:**

The present findings support the notion that both socio-economic status and lifestyle as well as psychosocial factors were related with good self-rated health. The interventions targeting these factors could improve the health status of the population. The depression was the most influential predictor of self-rated health, especially for the women and the elderly. Although lifestyle and psychosocial factors explained partly the the association between socio-economic status and health, the reason why socio-economic difference exists in health must be further explored. What’s more, it needs to be further studied why the same determinant has different influence strengths on the health of different groups of people.

## Introduction

Since the beginning of the 21st century, with the development of science and technology, people’s living standards have improved significantly. More and more people began to focus on health issues. In China, the overall health of the population has improved steadily. But the socio-economic difference in population’s health still exists. Therefore, it is significant to find the factors associated with the population’s health and to reduce the socio-economic difference in health in China. It is not only related to the quality of the population, but also related to economic development. Thus, this study aims to examine the determinants of self-rated health and to explore the process through which socio-economic status may impact self-rated health.

## Background

Self-rated health (SRH) is not only usually used to measure the health status of the population and health inequalities within that populations [1–3], but it is also used as a predictor of morbidity, mortality and health services utilization [4–6]. Literature from Western countries reports a socio-economic gradient in SRH with improvements in health status correlated with an increase in socio-economic status [7–9]. Other predictors, such as demographic characteristics, e.g. age, sex and marital status [10]; lifestyle factors, e.g. physical activity [11]; psychological factors, e.g. depression [12] and life satisfaction [13] have been shown to account for variations in SRH. However, some studies have found that for different groups of people, the impact factors of health may be different. For example, Damian et al. [14] found the effect of social class on health is markedly modified by age, which means social class was an important factor among the youngest group, but not a determinant among the oldest group.

While SRH has been widely used to assess population health and health inequalities in high-income countries, only a few studies have analyzed the health-related factors among Chinese population. Some scholars found gender was associated with health and women were less likely to report good health than men in China [15–17]. This may be related to the actual situation of Chinese families. In China, the lifestyle of women and men are different. Although more and more women choose to work outside home, the household related duties are still more dominant for women. Men mainly work outside the household and may not do housework. Education and income were often reported to be associated with people’s health, and people with higher level of education or annual income were more likely to have a good self-rated health [15, 16, 18]. Income seems to be able to provide financial support for heath. The findings by Liang et al. [17] suggested that variations in levels of depression were associated with variations in SDH for Chinese population. One study found rural residents tended to feel healthier compared to urban residents [15]. Although rural residents have low socio-economic status compared to urban residents, they were more likely to report good health. However, Pei and Rodriguez [19] found a strong relationship between residential affiliation and self-rated health, and those living in rural area were less likely to report good SRH. Evidence shows socio-economic disparities in health exist in China. Chen et al. [20] found significant socioeconomic status (SES) differences in the mean level of health. More and more studies focus on the socio-economic difference in health of the elderly. Beydoun and Popkin [21] found there were wide socio-economic disparities in the functional health of older adults in China. The study by Zhou [22] showed poor socio-economic status are negative impacts factors for the elderly living independently in rural China. They thought special attention is needed to those with lower socio-economic status and less children’s support. Sun et al. [16] used the EQ-5D instrument to measure the population health status in China, and found that health status declines with advancing age. It seems that vulnerable groups such as women, the elderly are less likely to report good health. Further research is needed to verify this, and to find out the reasons.

Theories have been put forward to explain socio-economic disparities in SRH [23]. Elstad [23] thought lifestyle factors may partly explain socio-economic disparities in SRH after controlling for individual risk factors, such as physical inactivity, obesity, and smoking. Psychosocial factors may also partly explain socio-economic disparities in SRH [23], because depression is more prevalent amongst those in low socio-economic status. However, none of these theories have been able to completely explain the socio-economic disparities in SRH and none of these theories have been examined in low- or middle- income countries.

In summary, further empirical studies regarding the determinants of health are needed, and the factors that affect the health of different groups have not been explored in China. In addition, to our knowledge, there is an absence of studies detailing the mechanism by which socio-economic status impacts SRH in China. The purpose of this study is to: 1) evaluate the factors that account for variations in SRH among Chinese citizens; and to 2) explore the process through which socio-economic status may impact self-rated health.

## Methods

### Data

Data were derived from the Chinese General Social Survey (CGSS) (2013), which was collected by the Department of Sociology at Renmin University of China and the Survey Research Center of Hong Kong University of Science and Technology in September and October, 2013. The Chinese General Social Survey was designed to understand the quality of life and health status of rural and urban families in China. The survey used a five-stage stratified sampling method (province, county, town, village and household), covering 28 provinces (municipalities),^1^ 134 counties (districts), 374 towns (streets), 491 villages (neighborhood committees), and 11,438 households, thereby yielding 11,438 useable respondents broadly representative of the whole of China. In a household, only one respondent was surveyed, and the response rate was 95.32%. Respondents were Chinese nationals aged 18 years or older. The CGSS contained detailed information on individual characteristics, such as age, sex, ethnicity, marital status, education, residence, household income, SRH etc. After excluding observations where there were missing values to pertinent variables, our analysis data set contained 9668 survey respondents, representing 84.5% of the original sample.

### Measures

#### Outcome measure: Self-rated health (SRH)

The dependent variable in the current study was the SRH of the survey respondents. SRH was evaluated by the question “How do you rate your health?”. Respondents chose their answers from a 5-point scale: very poor, poor, average, good and very good. In our analysis, SRH was dichotomized into two groups: good health, where SRH was either good or very good; and poor health, where SRH was very poor, poor or average [24, 25].

### Explanatory variables

Four sets of explanatory variables were used: Demographic characteristics, socio-economic status, lifestyle factors, and psychosocial factors.

### Demographic characteristics

Following the tradition of previous empirical studies, demographic characteristics included age, sex, ethnicity, marital status and place of residence, i.e. urban, suburban or rural [15, 26, 27]. Regional dummy variables were also included in our analysis. According to China’s economic development and administrative divisions, 28 provinces were divided into four categories, namely Eastern China (including 9 provinces),^2^ Middle China (including 6 provinces)^3^, Northeast China (including 3 provinces)^4^ and Western China (including 10 provinces)^5^.

### Socio-economic status

Objective and subjective measures were used to evaluate socio-economic status. Education, employment and annual household income were deemed as objective measures that have often been used in the literature to measure socio-economic status [12, 13]. In our study, education was divided into three classes: primary education (including primary or no education), secondary education (including middle or high school), and college or higher education. Employment status was categorized into non-farm work, farm work, and not working. Annual household income was captured as annual income in the year prior to the survey and was categorized into four classes (≤9999 RMB or ≤ 1640 dollars, 10,000-49,999 RMB or 1640–8201 dollars, 50,000-99,999 RMB or 8201–16,402 dollars, and ≥100,000 RMB or ≥16,402 dollars).

Subjective measures of socio-economic status included self-reported social class and socio-economic status compared with their peers. As there was an absence of correlation between these two subjective measures (Correlation coefficient < 0.3), both were included in the analysis. Subjective social class was assessed by asking “In our society, some people are in the upper classes of society, some people are in the lower classes of society, which level do you think you are in?”. Respondents self-rated scores of their social class on a 10-point scale. The scores were divided into three categories (low, score 1–3; middle, score 4–5; high, score 6–10) with higher scores indicating higher social class. Subjective socio-economic status compared with peers was assessed by asking “Compared with your peers, how do you perceive your own socio-economic status?” Respondents chose their answers from a four-point scale (1 = higher; 2 = similar; 3 = lower; 4 = hard to say).

### Lifestyle factors

Lifestyle factors included body mass index (BMI) and physical activity. BMI, which measures relative weight, was calculated from self-rated height and weight as weight divided by height squared (kg/m^2^). The respondent’s BMI was divided into four categories based on the guidelines for Asian populations’ BMI [28]: underweight (<18.5 kg/m^2^), normal weight (18.5–23 kg/m^2^), overweight (23–27.5 kg/m^2^) and obese (> = 27.5 kg/m^2^). The level of physical activity was determined with the question: “Over the past year, how often do you take exercise?” with the options ‘everyday’, ‘several times a week’, ‘several times a month’, ‘several times a year or less’, or ‘never’. We combined the respondents who reported ‘everyday’, ‘several times a week’, and ‘several times a month’ and coded them as being physically active. Other categories were coded as physically inactive.

### Psychosocial factors

We follow Perlman and Bobak [13] and Prus [29] who used self-reported life satisfaction as a psychosocial factor. This indicator was based on respondents’ description of their happiness in life and was based on responses to the question, “In general, do you think your life is happy or not?”. Respondents had an option to choose from a five-point scale ranging from ‘very unhappy’ to ‘very happy’. Life satisfaction was divided into three categories: unhappy (very unhappy and unhappy), average and happy (very happy and happy). The respondent’s relationship with others was also considered as a psychosocial factor and potentially correlated with SRH [30]. Respondents were asked “How would you describe relations with your families and friends?”, and the answers ranged from ‘very bad’ to ‘very good’ on a five-point scale. We created a three category variable, good relations, normal relations and bad relations, by combining those who responded ‘very bad’ and ‘bad’, and those who responded ‘very good’ and ‘good’. Depression was a further psychosocial factor that may influence SRH [12]. Depression was assessed with the question “In the past 4 weeks, how often do you feel depressed or frustrated?” with the respondent answering: ‘always’, ‘often’, ‘sometimes’, ‘seldom’, or ‘never’. Responses in the categories, ‘always’ and ‘often’, were combined and classified as severe depression. Those who responded, ‘seldom’ and ‘never’, were also combined and classified as having few or no depression and respondents who reported ‘sometimes’ was classified as having mild depression.

### Statistical analysis

Descriptive analysis of the dependent and explanatory variables was presented in Table 1. We then assessed the distribution of SRH across demographic, socio-economic, lifestyle, and psychosocial factors (Table 2). Pearson’s *X* ^2^ was used to examine the statistical significance between categorical variables [15, 17, 25]. A binary logistic regression model was used to analyze the determinants of SRH. Odds ratios (ORs) for good SRH were reported (Table 3). We initially assessed each potential determinant of good SRH through use of a univariate logistic regression model and reported the crude ORs for each variable (Model 1, Table 3). Then the multivariate analysis was run based on the result from univariate analysis. Through backwards stepwise logistic analysis, with a significance level of 0.10 to enter and to stay in the model, the multivariate model (Model 2, Table 3) including all significant variables which was derived from Model 1. The specific approach of backwards stepwise regression is as follows. The model involves starting with all significant variables in univariate analysis (in Model 1), then with the significance level of 0.10, deleting the variable (if any) whose loss gives the most statistically insignificant deterioration of the model fit, and repeating this process until no further variables can be deleted without a statistically significant loss of fit. Then we conduct sub-sample analysis, in which, we divided the sample according to different groups, such as age groups, gender and geographical areas. The results were showed in the Appendix 1. In order to examine the extent to which lifestyle and psychosocial factors explained the association between socio-economic status and SRH, we conducted another logistic regression. After controlling for demographic characteristics variables which were correlated with SRH in univariate analysis, we only controlled for socio-economic status variables in the baseline Model 3 (Table 4). After fitting Model 3, we added lifestyle variables to see if these lifestyle factors further explained the effect of socio-economic status on SRH (Model 4, Table 4). Additionally, after fitting Model 3, we added the psychosocial factors to see if the psychosocial factors were also important in accounting for the association between socio-economic status and SRH (Model 5, Table 4).

**Table 1:** Descriptive statistic of the total study population (*N* = 9,668)

Variables	Category	Number	Percent
Dependent variable			
Self-rated health	Good SRH	6,285	65.01
	Poor SRH	3,383	34.99
Independent variables			
Demographic characteristics			
Gender	Female	4,724	48.86
	Male	4,944	51.14
Age group	18–29	1,161	12.01
	30–39	1,645	17.01
	40–49	2,119	21.92
	50–59	1,911	19.77
	60–69	1,635	16.91
	70+	1,197	12.38
Ethnicity	Other ethnic minorities	786	8.13
	Han nationality	8,882	91.87
Marital status	Divorced, separated, or widowed	970	10.03
	Married	7,812	80.80
	Single	886	9.16
Residence	Rural	3,779	39.09
	Urban	3,549	36.71
	Suburban	2,340	24.20
Regions	West	2,446	25.30
	East	3,536	36.57
	Middle	2,337	24.17
	Northeast	1,349	13.95
Socio-economic status			
Education degree	Primary or no education	3,352	34.67
	Secondary education	4,746	49.09
	College or higher	1,570	16.24
Work status	Not working	3,451	35.70
	Non-farm work	4,024	41.62
	Farm work	2,193	22.68
Household Income (RMB/dollars)	≤¥9,999 (or ≤ $ 1,640)	980	10.14
	¥10,000–49,999 (or $1,640–8,201)	4,399	45.50
	¥50,000–99,999 (or $8,201–16,402)	2,867	29.65
	≥¥100,000 (or ≥ $16,402)	1,422	14.71
Self-rated social class	Low (score 1–3)	2,863	29.61
	Middle (score 4–5)	6,056	62.64
	High (score 6–10)	749	7.75
Socio-economic status compared with peers	Lower	3,318	34.32
	Higher	450	4.65
	Similar	5,525	57.15
	Hard to say	375	3.88
Lifestyle factors			
BMI	Normal weight	6,698	69.28
	Underweight	833	8.62
	Over weight	1,911	19.77
	Obese	226	2.34
Physical activity	Inactive	6,775	70.08
	Active	2,893	29.92
Psychosocial factors			
Life satisfaction	Happy life	7,071	73.14
	Unhappy life	852	8.81
	Average	1,745	18.05
Depression	Severe	770	7.96
	Mild	1,937	20.04
	Few or no	6,961	72.00
Relations with families and friends	Good	5,029	52.02
	Poor	1,293	13.37
	Normal	3,346	34.61

**Table 2:** The distribution of SRH across socio-demographic factors, socio-economic status, lifestyle factors and psychosocial factors (*N* = 9,668)

Variables	Category	Good SRH		*X* ^2^	P
		N	%		
Demographic characteristics					
Gender	Female	2,952	62.49	25.77	<0.0001
	Male	3,333	67.42		
Age group	18–29	1,012	87.17	1100.00	<0.0001
	30–39	1,354	82.31		
	40–49	1,509	71.21		
	50–59	1,160	60.70		
	60–69	786	48.07		
	70+	464	38.76		
Ethnicity	Other ethnic minorities	502	63.87	0.49	0.48
	Han nationality	5,783	65.11		
Marital status	Divorced, separated, or widowed	454	46.80	250.49	<0.0001
	Married	5,107	65.37		
	Single	724	81.72		
Residence	Rural	2,235	59.14	95.99	<0.0001
	Urban	2,467	69.51		
	Suburban	1,583	67.65		
Regions	West	1,462	59.77	123.38	<0.0001
	East	2,539	71.80		
	Middle	1,411	60.38		
	Northeast	873	64.71		
Socio-economic status					
Education degree	Primary or no education	1,668	49.76	573.92	<0.0001
	Secondary education	3,354	70.67		
	College or higher	1,263	80.45		
Work status	Not working	1797.00	52.07	698.44	<0.0001
	Non-farm work	3218.00	79.97		
	Farm work	1270.00	57.91		
Household Income (RMB/dollars)	≤¥9,999 (or ≤ $ 1,640)	375.00	38.27	541.96	<0.0001
	¥10,000–49,999 (or $1,640–8,201)	2680.00	60.92		
	¥50,000–99,999 (or $8,201–16,402)	2141.00	74.68		
	≥¥100,000 (or ≥ $16,402)	1089.00	76.58		
Self-rated social status	Low (score 1–3)	1,529	53.41	243.78	<0.0001
	Middle (score 4–5)	4,211	69.53		
	High (score 6–10)	545	72.76		
Socio-economic status compared with peers	Lower	1,785	53.80	286.80	<0.0001
	Higher	343	76.22		
	Similar	3,904	70.66		
	Hard to say	253	67.47		
Lifestyle factors					
BMI	Normal weight	4478.00	66.86	95.94	<0.0001
	Underweight	421.00	50.54		
	Over weight	1260.00	65.93		
	Obese	126.00	55.75		
Physical activity	Inactive	4,232	62.46	64.38	<0.0001
	Active	2,053	70.96		
Psychosocial factors					
Life satisfaction	Happy life	4,913	69.48	285.83	<0.0001
	Unhappy life	366	42.96		
	Average	1,006	57.65		
Depression	Severe	188	24.42	1500.00	<0.0001
	Mild	777	40.11		
	Few or no	5,320	76.43		
Relations with relatives and friends	Good	3,568	70.95	218.88	<0.0001
	Poor	648	50.12		
	Normal	2,069	61.84

**Table 3:** Determinants of health in China: odds ratios for good self-rated health (SRH)

Variables	Category	Model 1: Univariate analysis		Model 2: Multivariate analysis	
		OR	95% CI	OR	95% CI
Demographic characteristics					
Gender	Female	1.00		1.00	
	Male	1.24***	(1.14–1.35)	1.19***	(1.07–1.32)
Age group	18–29	1.00		1.00	
	30–39	0.69***	(0.55–0.85)	0.63***	(0.48–0.82)
	40–49	0.36***	(0.30–0.44)	0.38***	(0.29–0.49)
	50–59	0.23***	(0.19–0.28)	0.24***	(0.18–0.31)
	60–69	0.14***	(0.11–0.17)	0.16***	(0.12–0.21)
	70+	0.09***	(0.08–0.11)	0.11***	(0.09–0.15)
Ethnicity	Other ethnic minorities	1.00			
	Han nationality	1.06	(0.91–1.23)		
Marital status	Divorced, separated, or widowed	1.00		1.00	
	Married	2.15***	(1.88–2.45)	0.81**	(0.68–0.95)
	Single	5.08***	(4.11–6.28)	0.75*	(0.55–1.02)
Residence	Rural	1.00			
	Urban	1.58***	(1.43–1.73)		
	Suburban	1.44***	(1.30–1.61)		
Regions	West	1.00		1.00	
	East	1.71***	(1.54–1.91)	1.28***	(1.11–1.47)
	Middle	1.03	(0.91–1.15)	1.11	(0.96–1.27)
	Northeast	1.23***	(1.08–1.42)	1.15*	(0.98–1.36)
Socio-economic status					
Education degree	Primary or no education	1.00			
	Secondary education	2.43***	(2.22–2.67)		
	College or higher	4.15***	(3.60–4.79)		
Work status	Not working	1.00		1.00	
	Non-farm work	3.67***	(3.32–4.07)	1.96***	(1.71–2.24)
	Farm work	1.27***	(1.14–1.41)	1.39***	(1.21–1.59)
Household Income (RMB/dollars)	≤¥9,999 (or ≤ $ 1,640)	1.00		1.00	
	¥10,000–49,999 (or $1,640–8,201)	2.52***	(2.18–2.90)	1.21**	(1.02–1.43)
	¥50,000–99,999 (or $8,201–16,402)	4.76***	(4.08–5.55)	1.46***	(1.20–1.77)
	≥¥100,000 (or ≥ $16,402)	5.28***	(4.42–6.30)	1.06	(0.84–1.34)
Self-rated social class	Low (score 1–3)	1.00		1.00	
	Middle (score 4–5)	1.99***	(1.82–2.18)	1.20***	(1.07–1.35)
	High (score 6–10)	2.33***	(1.95–2.78)	1.26**	(1.02–1.57)
Socio-economic status compared with peers	Lower	1.00		1.00	
	Higher	2.75***	(2.19–3.46)	1.89***	(1.45–2.48)
	Similar	2.07***	(1.89–2.26)	1.31***	(1.16–1.46)
	Hard to say	1.78***	(1.42–2.23)	1.11	(0.84–1.45)
Lifestyle factors					
BMI	Normal weight	1.00		1.00	
	Underweight	0.51***	(0.44–0.59)	0.68***	(0.57–0.82)
	Over weight	0.96	(0.86–1.07)	0.92	(0.81–1.04)
	Obese	0.62***	(0.48–0.82)	0.59***	(0.43–0.82)
Physical activity	Inactive	1.00		1.00	
	Active	1.47***	(1.37–1.61)	1.16**	(1.03–1.31)
Psychosocial factors					
Life satisfaction	Happy life	1.00		1.00	
	Unhappy life	0.33***	(0.29–0.38)	0.68***	(0.57–0.82)
	Average	0.60***	(0.54–0.67)	0.75***	(0.66–0.85)
Depression	Severe	1.00		1.00	
	Mild	2.07***	(1.72–2.50)	1.86***	(1.51–2.29)
	Few or no	10.04***	(8.44–11.94)	7.89***	(6.50–9.57)
Relations with relatives and friends	Good	1.00		1.00	
	Poor	0.41***	(0.36–0.47)	0.74***	(0.63–0.86)
	Normal	0.66***	(0.60–0.73)	0.79***	(0.71–0.88)
Pseudo R^2^				0.2305	

Multivariate model including all significant variables through backwards stepwise logistic analysis

*OR* odds ratio, *95% CI* 95% Confidence Intervals

*** *p* < 0.01, ** *p* < 0.05, * *p* < 0.1

**Table 4:** Examine the extent to which lifestyle or psychosocial factors explained the effect of socio-economic status on good SRH

Variables	Category	Model 3		Model 4		Model 5	
		OR	95% CI	OR	95% CI	OR	95% CI
Demographic characteristics							
Gender	Female	1.00		1.00		1.00	
	Male	1.18***	(1.07–1.30)	1.18***	(1.07–1.29)	1.18***	(1.07–1.30)
Age group	18–29	1.00		1.00		1.00	
	30–39	0.57***	(0.44–0.73)	0.55***	(0.43–0.71)	0.65***	(0.49–0.84)
	40–49	0.33***	(0.26–0.43)	0.32***	(0.25–0.41)	0.40***	(0.31–0.52)
	50–59	0.22***	(0.17–0.28)	0.21***	(0.16–0.27)	0.25***	(0.19–0.32)
	60–69	0.16***	(0.13–0.21)	0.16***	(0.12–0.21)	0.16***	(0.13–0.22)
	70+	0.13***	(0.10–0.17)	0.13***	(0.10–0.17)	0.13***	(0.09–0.17)
Marital status	Divorced, separated, and widowed	1.00		1.00		1.00	
	Married	0.98	(0.84–1.15)	0.97	(0.83–1.14)	0.81**	(0.68–0.95)
	Single	0.86	(0.64–1.14)	0.83	(0.62–1.10)	0.80	(0.95–1.08)
Residence	Rural	1.00		1.00		1.00	
	Urban	1.04	(0.91–1.20)	1.00	(0.87–1.15)	1.07	(0.92–1.24)
	Suburban	1.06	(0.92–1.21)	1.03	(0.90–1.18)	1.13	(0.97–1.30)
Regions	West	1.00		1.00		1.00	
	East	1.44***	(1.27–1.65)	1.43***	(1.25–1.62)	1.44***	(1.27–1.63)
	Middle	1.12*	(0.98–1.27)	1.12*	(0.99–1.28)	1.09	(0.95–1.26)
	Northeast	1.29***	(1.11–1.51)	1.29***	(1.11–1.50)	1.15	(0.97–1.36)
Socio-economic status							
Education degree	Primary or no education	1.00		1.00		1.00	
	Secondary education	1.26***	(1.13–1.42)	1.25***	(1.11–1.40)	1.19	(1.06–1.35)
	College or higher	1.09	(0.91–1.32)	1.06	(0.87–1.28)	1.05	(0.86–1.28)
Work status	Not working	1.00		1.00		1.00	
	Non-farm work	1.93***	(1.70–2.19)	1.96***	(1.73–2.23)	1.93***	(1.69–2.22)
	Farm work	1.53***	(1.32–1.76)	1.56***	(1.35–1.80)	1.45***	(1.25–1.70)
Household Income (RMB/dollars)	≤¥9,999 (or ≤ $ 1,640)	1.00		1.00		1.00	
	¥10,000–49,999 (or $1,640–8,201)	1.35***	(1.15–1.58)	1.31***	(1.12–1.54)	1.20**	(1.01–1.42)
	¥50,000–99,999 (or $8,201–16,402)	1.76***	(1.46–2.11)	1.69***	(1.41–2.03)	1.46***	(1.20–1.78)
	≥¥100,000 (or ≥ $16,402)	1.37***	(1.09–1.70)	1.31**	(1.05–1.64)	1.07	(0.85–1.36)
Self-rated social status	Low (score 1–3)	1.00		1.00		1.00	
	Middle (score 4–5)	1.39***	(1.25–1.55)	1.38***	(1.24–1.54)	1.22***	(1.08–1.37)
	High (score 6–10)	1.42***	(1.16–1.74)	1.39***	(1.13–1.70)	1.31**	(1.05–1.63)
Socio-economic status compared with peers	Lower	1.00		1.00		1.00	
	Higher	2.63***	(2.04–3.39)	2.60***	(2.01–3.35)	1.90***	(1.45–2.49)
	Similar	1.70***	(1.53–1.88)	1.68***	(1.51–1.87)	1.32***	(1.17–1.48)
	Hard to say	1.22	(0.95–1.57)	1.19	(0.93–1.53)	1.13	(0.86–1.48)
Lifestyle factors							
BMI	Normal weight			1.00			
	Underweight			0.66***	(0.56–0.79)		
	Over weight			0.94	(0.84–1.06)		
	Obese			0.59***	(0.44–0.80)		
Physical activity	Inactive			1.00			
	Active			1.17***	(1.04–1.31)		
Psychosocial factors							
Life satisfaction	Happy life					1.00	
	Unhappy life					0.68***	(0.57–0.82)
	Average					0.75***	(0.66–0.85)
Depression	Severe depression					1.00	
	Mild depression					1.86***	(1.51–2.29)
	Less or no					7.90***	(6.51–9.59)
Family relations	Good					1.00	
	Poor					0.73***	(0.63–0.85)
	Normal					0.79***	(0.71–0.88)
Pseudo R^2^		0.1428		0.2101		0.2290	

Model 3 controlled for demographic characteristics (except ethnicity variable, which was not correlated with SRH in univariate analysis) and socio-economic status factors

Model 4 also controlled lifestyle factors in addition to the factors controlled in the baseline Model 3

Model 5 also controlled for psychosocial factors in addition to the factors controlled in baseline Model 3

OR, odds ratio; 95% CI, 95% Confidence Intervals

***p < 0.01, **p < 0.05, *p < 0.1

All of the results were presented as ORs, and 95% confidence intervals (CIs). An ORs above one indicates that the specified determinant was more likely to report good SRH. All analyses were performed using Stata, version 13.0.

## Results

### Characteristic of the sample

Table 1 presents the descriptive statistic for the study variables. About 65% of the survey respondents reported good SRH. The sample was almost equally divided between men and women (51.14% vs 48.86%). Nearly half of the respondents were aged 50 or over (49.06%). Survey respondents were mostly of Han nationality (91.87%), married (80.80%) and from urban or suburban regions (60.91%). About 50% of the respondents had secondary education and 42% of the respondents were engaged in non-farm work. Most respondents (75.15%) reported that their annual household income was between 10,000 and 99,999 RMB (or between 1,640 and 8,201 dollars). More than half of the respondents perceived their social class as “middle” (62.64%) and thought that their socio-economic status was similar to their peers (57.15%). More than half of the respondents were of normal weight (69.28%), physically inactive (70.08%), reported a happy life (73.14%), felt less or not depressed (72.00%) and had a good relationship with their families and friends (52.02%).

### Distribution of SRH

Table 2 presents the distribution of SRH once stratified by each potential explanatory variable for each of the four groups of factors. The *X* ^2^ test was used to assess whether there were significant differences in SRH for each set of explanatory variable. The only variable that was not significant was ethnicity. Men (67.42%), the younger (87.17% for 18–29), single respondents (81.72%), those living in urban regions (69.51%) and coming from Eastern China (71.80%) reported significantly good SRH. Compared with primary or no education (49.76%), the prevalence of good SRH associated with secondary education (70.67%) or college or higher education (80.45%) was clearly higher. Respondents with good SRH were more likely to be those who were engaged in non-farm work (79.97%), had household income more than 100,000 RMB (or 16,402 dollars) (76.58%), were in high social classes (72.76%) and had higher socio-economic status compared with their peers (76.22%). Good SRH was associated with those who reported being normal weight (66.86%) and physically active (70.96%). The prevalence of good SRH was greater in the respondents who reported a happy life, felt less or not depressed and had good relationships with families and friends (69.48, 76.43 and 70.95% respectively).

### Determinants of SRH

Table 3 presents the binary logistic regression results. The univariate logistic regression analysis suggested that all the potential variables except ethnicity were significantly associated with SRH (Model 1). Men, married or single respondents, urban or suburban residents and those living in Eastern China tended to report good SRH. Age was negatively related to good SRH. Those with a higher level of education or household income, being engaged in non-farm or farm work, being in high social class and higher socio-economic status compared with their peers were more likely to report good SRH. Respondents who were underweight or obese, reported an unhappy life and had a poor relationship with families and friends were less likely to report good SRH. Physically active respondents and those felt less or not depressed tended to report good SRH.

The multivariate model was based on the application of backwards stepwise logistical regression analysis (Model 2, Table 3). Men (OR 1.19; 95% CI 1.07–1.32) and those living in Eastern China (OR 1.28; 95% CI 1.11–1.47) were more likely to report good SRH. The elderly (OR 0.11; 95% CI 0.09–0.15 for 70+) and married (OR 0.81; 95% CI 0.68–0.95) or single (OR 0.75; 95% CI 0.55–1.02) respondents were less likely to report good SRH. Respondents who were engaged in non-farm work (OR 1.96; 95% CI 1.71–2.24) and farm work (OR 1.39; 95% CI 1.21–1.59) were more likely to report good SRH. High household income was associated with good SRH. Good SRH was significantly associated with high social class (OR 1.26; 95% CI 1.02–1.57) or higher social-economic status compared with peers (OR 1.89; 95% CI 1.45–2.48). There was an inverted U-shaped relation between BMI and good SRH. Underweight (OR 0.68; 95% CI 0.57–0.82) or obese (OR 0.59; 95% CI 0.43–0.82) respondents had a lower chance of reporting good SRH. Good SRH was more common among respondents who were physically active (OR 1.16; 95% CI 1.03–1.31) and felt less or not depressed (OR 7.89; 95% CI 6.50–9.57), but was less common among respondents who reported an unhappy life (OR 0.68; 95% CI 0.57–0.82), and had poor relationships with families and friends (OR 0.74; 95% CI 0.63–0.86).

### Sub-sample analysis

When we divided the sample into sub-samples by age group, gender and geographical area, the results were robust. We showed the the results of the sub-sample analysis in the Appendix 1 in order to save space. Table 5 in Appendix 1 shows the results of the analysis stratified by age group. Depression played an important role in the SRH of the elderly. For example, the respondents who felt less or not depressed (OR 10.98; 95% CI 7.19–16.76) wore more likely to report good SRH. It was worth noting that physical activity only impacted the SRH of the elderly. Table 6 in Appendix 1 presents the results of the analysis stratified by gender. In general, the effect of socio-economic factors on SRH for men was much stronger than for women. But the effect of psychological factors such as depression on women’s SRH was stronger. Table 7 in Appendix 1 reports the results of the analysis once stratified by geographical area. Income was an important determinant for the good SRH of respondents living in the Middle and West China, while depression was an important factor to affect SRH of respondents in the East China.

### Mediating mechanism of socio-economic status on good SRH

Table 4 shows the extent to which the association between socio-economic status and good SRH was explained by lifestyle or psychosocial factors. When lifestyle factors were added to the models, while the effect of demographic characteristics on SRH hardly changed, the relationship between the socio-economic status (e.g. secondary education, household income, social class and socio-economic status compared with peers) and SRH attenuated somewhat, but still remained strong and significant (Model 4). For example, the relative risk of high social class on good SRH was 1.42 (95% CI 1.16–1.74) in the base Model 3, and 1.39 (95% CI 1.13–1.70) in Model 4 after controlling for lifestyle factors. In addition, when psychosocial factors were added to the models, while the influence of demographic characteristics on SRH remained nearly the same or increased, the association between secondary education and household income (≥100,000 RMB or ≥16,402 dollars), and SRH became insignificant (Model 5). This result suggests that psychosocial factors completely explained the impact of secondary education and household income (≥100,000 RMB or ≥16,402 dollars) on SRH. The association between social class and social-economic status compared with peers, and good SRH were largely explained by psychosocial factors (Model 5). For example, compared to Model 3, the ratio of social-economic status compared with peers in Model 5 fell from 2.63 to 1.90, 1.70 to 1.32 for “higher” and “similar” respectively.

We also used the data from the Chinese General Social Survey (CGSS) (2010) to conduct robust test, and the results were showed in the Appendix 2. Though we added smoking habit variable as a lifestyle factor, smoking habit was not a determinant of SRH in the multivariate analysis. All the other results are robust.

## Discussion

This study examined the determinants of SRH in China and aimed to identify health-related inequalities in a representative sample of 9,668 households. We also explored the mediating mechanisms identified in the relationship between socio-economic status and SRH. To our knowledge, there are few studies that assess the determinants of SRH in the Chinese population and there is an absence of studies that investigate the mediating mechanisms by which socio-economic status impacts SRH among the Chinese population. Our study provides evidence of the association of SRH with several demographic factors, socio-economic factors, lifestyle factors and psychosocial factors. Once our analysis was stratified by age group, gender and geographical area, the results were robust. But for different groups, some factors showed stronger effects, while some showed weaker influence. We also found that lifestyle factors and psychological factors explained only a modest portion of the association between socio-economic status and SRH. We use data from another survey year (2010) to conduct robust test, and the results are consistent.

Almost two thirds of the respondents in our study reported good SRH. Significant differences were observed in SRH across most of the demographic characteristics. We found women and the elderly were less likely to report good SRH. The findings may derive from some factors: unfavorable biological characteristics, greater propensity to admit illness, or different social roles that make women more vulnerable to poor SRH. The elderly is more susceptible to disease, which leads to poor health.

Previous studies have found a strong association between socio-economic status and SRH [12, 29, 31, 32]. We have calculated the concentration index based on the survey data, and found it was 0.3095. That indicates the rich had a greater share of good-health in China. Therefore, income-related health inequality exists in China. In our study, employment status was positively associated with SRH, which was consistent with other study [33]. Psychological factors may partly explain why people who work tend to report better SRH. Indeed, there was some evidence of this in our data, since the relationship between farm work and SRH attenuated somewhat when psychological factors were adjusted (Model 6, Table 4). High education [19, 29, 34, 35] and income [13, 25] was often reported to be related to good SRH. In our analysis, the effect of education on SRH was modified by psychological factors. People with high household income not only have a greater chance to gain access to health care [36], but also can afford the expensive commercial insurance. The positive relationship between income and good SRH was much stronger among men and those living in Middle and West China. This may be due to the fact that men are more concerned with income than women, and income is guaranteed for good health of individuals living in undeveloped areas. Subjective social class and social-economic status compared with peers were positively associated with SRH. Though people’s lifestyle factors played a role in the association between socio-economic status and SRH, they did not completely explain the association. Socio-economic disparities in SRH may be best understood as multi-causal, generating from the impact of many factors [37].

Lifestyle factors were strongly and independently associated with SRH. In particular, physical activity is essential for the health of the elderly. The literature has demonstrated that an inactive lifestyle and poor diet not only increase the risk of death and the associated lost years of life, they also are associated with high rates of disability, and lower quality of life [38–43]. An inverted U-shaped relationship between BMI and good SRH was found in our study, which is similar to the findings by Østbye [44] and Molarius [45]. People who were underweight and obese were less likely to report good SRH. Underweight may be due to malnutrition and obesity is a significant risk factors for ill health. Both malnutrition and illness are negatively related to good health. In the robust test, we found smoking habit was not the determinant of health, which was consistent with Gilmore [30]. Adding the smoking habit variable did not alter the results of the present study.

Psychosocial factors were also strong predictors of SRH. Greater life satisfaction was significantly associated with better SRH [13]. The strongest predictor of SRH in our study was depression, especially for women and the elderly, which was consistent with previous studies [12, 46, 47]. Therefore, the depression much be considered for when dealing with health problems, especially for women and the elderly. Good relations with families and friends are good for health. In China, relationships are important social networks. Good relationships with families and friends not only help people in advancing their education and employment opportunities, it also helps reduce stress. Similar findings were also found in Russia [48] and Ukraine [30] where family relations as social networks had positive impacts on SRH by moderating stress.

The study also implies that the association between socio-economic status and good SRH was partly explained by lifestyle and psychosocial factors. People who have different socio-economic status have different lifestyles. It may be that those with higher socio-economic status are more inclined to participate in fitness activities [45], which may be because of their adequate finance or willingness. It is expected that people will be more likely to report stress and depression when they are in lower socio-economic status. The attenuation of the socio-economic effects when lifestyle and psychosocial factors were considered suggests that these factors do explain some of the association between socio-economic status and SRH. However, because most socio-economic status variables remained significant after taking lifestyle and psychosocial factors into account, the findings suggest that the lifestyle and psychosocial factors considered in the current study are not likely to be the dominant mechanism by which socio-economic status impacts SRH. Our findings were consistent with Lantz et al. and Lynch et al. [37, 49]. But those authors ignored psychosocial factors as their mediating mechanism.

The current study had several potential limitations. First, because we used cross-sectional data, we were unable to verify the strict causal relationship between the explanatory variables and SRH. For example, it was not clear whether people were unemployed because of their poor health. However, when we measured the four groups of potential factors, we used several different explanatory variables as the potential determinants of SRH in each group, and the influence of these variables on SRH were consistent. It was less likely that there was a causality relationship between SRH and all the explanatory variables in each group. For example, SRH cannot affect the last year’s household income. Besides, a set of factors independently correlated with SRH in our study were also found to be determinants of good SRH in other longitudinal studies [44, 50–52]. We were also unable to take into account the changes in health, socioeconomic characteristics, lifestyles and psychological factors over time due to the data limit. Future research is needed to address this issue. Second, we were unable to examine the impact of all the potential predictor variables. However, we included as many variables as possible. Third, many variables such as the socio-economic factors (e.g. subjective social class in society and social-economic status compared with peers), life satisfaction and SRH were subjective. These were prone to participant response bias. However, the way the questions were asked were similar to previous studies [14, 19, 24, 25, 29, 32], and these subjective measures have been found to be valid in those studies. So potential response bias may be small. Fourth, although we tried to find mediating mechanisms in the relationship between socio-economic status and SRH, due to limitations associated with the variables included in the survey, we were only able to assess the role of lifestyle and psychosocial factors. This is clearly an opportunity for further research as well as to investigate other links between SRH and various political, economic, and social processes [53, 54].

## Conclusion

Researching the determinants of health has important implications for the development of public health policy. A greater understanding of the potential for interventions focused on the lifestyle and psychological factors of individuals to reduce socio-economic difference in health is needed. This study complements the international literature by reporting results from China, an upper-middle-income country, that highlights the factors associated with variations in SRH and explores the processes through which socio-economic status impacts SRH. The findings suggest that socio-economic status, lifestyle and psychosocial factors were associated with SRH. Few or no depression was the most influential predictor of good SRH, which suggests that regulating emotions is important for good health, especially for the elderly, women and respondents from East China. Physical activity is an important determinant among the elderly group. Income plays an important role in the health of men and those respondents coming from Middle or West China. Our findings also suggest that the association between socio-economic status and good SRH is partly explained by lifestyle and psychosocial factors. However, the mediating mechanism of the effect of the socio-economic status on good SRH needs to be further explored. Consideration of the mediating mechanism that can explain socio-economic disparities in SRH is an important issue for public health policy. To answer questions of why socio-economic disparities in health exist, the historical, political, economic, and social factors need to be explored. Overall, our findings suggest that interventions targeting socio-economic status, lifestyle and psychosocial factors could improve SRH for the population.

## Appendix 1

### Sub-sample analysis

**Table 5:** Determinants of good SRH by age group

Variables	Category	18–39 (*n* = 2806)		40–59 (*n* = 4030)		60+ (*n* = 2832)	
		OR	95% CI	OR	95% CI	OR	95% CI
Demographic characteristics							
Gender	Female			1.00			
	Male			1.20**	(1.02–1.40)		
Marital status	Divorced, separated, and widowed	1.00					
	Married	0.30**	(0.12–0.75)				
	Single	0.35**	(0.14–0.90)				
Regions	West	1.00		1.00			
	East	1.35**	(1.01–1.80)	1.29**	(1.04–1.60)		
	Middle	1.24	(0.90–1.72)	1.03	(0.84–1.27)		
	Northeast	1.21	(0.85–1.75)	0.99	(0.77–1.26)		
Socio-economic status							
Education degree	Primary or no education			1.00		1.00	
	Secondary education			1.21**	(1.02–1.45)	1.21**	(1.00–1.46)
	College or higher			1.06	(0.76–1.46)	0.97	(0.68–1.39)
Work status	Not working	1.00		1.00		1.00	
	Non-farm work	1.25*	(0.96–1.64)	2.48***	(2.02–3.03)	2.06***	(1.52–2.80)
	Farm work	0.80	(0.56–1.15)	1.75***	(1.41–2.17)	1.60***	(1.30–1.96)
Household Income (RMB/dollars)	≤¥9,999 (or ≤ $ 1.640)			1.00			
	¥10,000–49,999 (or $1,640–8,201)			1.48***	(1.09–1.99)		
	¥50,000–99,999 (or $8,201–16,402)			2.10***	(1.51–2.93)		
	≥¥100,000 (or ≥ $16,402)			1.57**	(1.06–2.33)		
Self-rated social class	Low (score 1–3)			1.00			
	Middle (score 4–5)			1.26***	(1.06–1.50)		
	High (score 6–10)			1.39*	(0.98–1.98)		
Socio-economic status compared with peers	Lower	1.00		1.00			
	Higher	2.67**	(1.18–6.07)	2.07***	(1.26–3.42)	1.64***	(1.15–2.34)
	Similar	1.47***	(1.16–1.87)	1.33***	(1.13–1.58)	1.18*	(0.97–1.42)
	Hard to say	2.17***	(1.25–3.76)	0.78	(0.52–1.18)	0.88	(0.52–1.50)
Lifestyle factors							
BMI	Normal weight	1.00		1.00			1
	Underweight	0.64**	(0.45–0.90)	0.64**	(0.45–0.90)	0.64***	(0.49–0.84)
	Over weight	0.76*	(0.55–1.03)	0.96	(0.80–1.15)	0.95	(0.77–1.17)
	Obese	0.48**	(0.25–0.92)	0.63*	(0.39–1.01)	0.61*	(0.34–1.09)
Physical activity	Inactive					1.00	
	Active					1.33***	(1.09–1.61)
Psychosocial factors							
Life satisfaction	Happy life	1.00		1.00		1.00	
	Unhappy life	0.60***	(041–0.88)	0.70***	(0.54–0.91)	0.74*	(0.53–1.05)
	Average	0.72**	(0.54–0.95)	0.77***	(0.63–0.93)	0.76**	(0.60–0.96)
Depression	Severe	1.00		1.00			
	Mild	2.10***	(1.39–3.19)	1.65***	(1.23–2.21)	2.46***	(1.57–3.86)
	Few or no	7.50***	(5.07–11.09)	7.19***	(5.48–9.43)	10.98***	(7.19–16.76)
Relations with families and friends	Good	1.00		1.00		1.00	
	Poor	0.60***	(0.41–0.85)	0.73***	(0.58–0.91)	0.78**	(0.62–0.99)
	Normal	0.78**	(0.61–0.99)	0.76***	(0.64–0.90)	0.85*	(0.70–1.02)
Pseudo R^2^		0.1300		0.1895		0.1446	

Multivariate model including all significant variables through backwards stepwise logistic analysis

*OR* odds ratio; 95% CI, 95% Confidence Intervals

****p* < 0.01, ***p* < 0.05, **p* < 0.1

**Table 6:** Determinants of good SRH by gender

Variables	Category	Male (*n* = 4944)		Female (*n* = 4724)	
		OR	95% CI	OR	95% CI
Demographic characteristics					
Age group	18–29	1.00		1.00	
	30–39	0.62***	(0.44–0.87)	0.71*	(0.49–1.03)
	40–49	0.43***	(0.32–0.59)	0.41***	(0.28–0.58)
	50–59	0.29***	(0.21–0.39)	0.23***	(0.16–0.32)
	60–69	0.19***	(0.14–0.26)	0.17***	(0.12–0.25)
	70+	0.15***	(0.11–0.22)	0.11***	(0.08–0.17)
Marital status	Divorced, separated, and widowed			1.00	
	Married			0.77**	(0.61–0.96)
	Single			0.91	(0.55–1.48)
Regions	West	1.00		1.00	
	East	1.32***	(1.08–1.61)	1.22**	(1.00–1.50)
	Middle	1.10	(0.91–1.34)	1.10	(0.90–1.35)
	Northeast	1.13	(0.90–1.42)	1.15	(0.90–1.46)
Socio-economic status					
Education degree	Primary or no education			1.00	
	Secondary education			1.25**	(1.05–1.48)
	College or higher			1.17	(0.86–1.58)
Work status	Not working	1.00		1.00	
	Non-farm work	2.19***	(1.79–2.69)	1.73***	(1.43–2.11)
	Farm work	1.68***	(1.36–2.07)	1.27**	(1.04–1.54)
Household Income (RMB/dollars)	≤¥9,999 (or ≤ $ 1.640)	1.00		1.00	
	¥10,000–49,999 (or $1,640–8,201)	1.46***	(1.15–1.84)	0.96	(0.75–1.24)
	¥50,000–99,999 (or $8,201–16,402)	1.80***	(1.38–2.36)	1.13	(0.85–1.50)
	≥¥100,000 (or ≥ $16,402)	1.43**	(1.03–1.98)	0.74*	(0.53–1.03)
Self-rated social class	Low (score 1–3)	1.00		1.00	
	Middle (score 4–5)	1.23**	(1.04–1.45)	1.16*	(0.98–1.37)
	High (score 6–10)	1.28	(0.94–1.75)	1.25	(0.91–1.70)
Socio-economic status compared with peers	Lower	1.00		1.00	
	Higher	1.99***	(1.36–2.90)	1.78***	(1.21–2.63)
	Similar	1.28***	(1.08–1.50)	1.34***	(1.14–1.57)
	Hard to say	0.88	(0.61–1.27)	1.45*	(0.96–2.18)
Lifestyle factors					
BMI	Normal weight	1.00		1.00	
	Underweight	0.60***	(0.45–0.80)	0.75**	(0.59–0.95)
	Over weight	0.89	(0.74–1.06)	0.97	(0.80–1.16)
	Obese	0.61**	(0.38–0.97)	0.57**	(0.37–0.89)
Physical activity	Inactive	1.00			
	Active	1.22**	(1.03–1.44)		
Psychosocial factors					
Life satisfaction	Happy life	1.00		1.00	
	Unhappy life	0.65***	(0.50–0.83)	0.72**	(0.55–0.94)
	Average	0.73***	(0.61–0.88)	0.77***	(0.64–0.93)
Depression	Severe	1.00		1.00	
	Mild	1.63***	(1.22–2.19)	2.14***	(1.59–2.87)
	Few or no	6.51***	(4.96–8.56)	9.66***	(7.31–12.77)
Relations with families and friends	Good	1.00		1.00	
	Poor	0.73***	(0.59–0.91)	0.75***	(0..60–0.93)
	Normal	0.77***	(0.66–0.90)	0.81**	(0.70–0.95)
Pseudo R^2^		0.2297		0.2340	

Multivariate model including all significant variables through backwards stepwise logistic analysis

*OR* odds ratio, *95% CI* 95% Confidence Intervals

****p* < 0.01, ***p* < 0.05, **p* < 0.1

**Table 7:** Determinants of good SRH by geographical area

Variables	Category	East (*n* = 3536)		Middle (*n* = 2337)		Northeast (*n* = 1349)		West (*n* = 2446)	
		OR	95% CI	OR	95% CI	OR	95% CI	OR	95% CI
Demographic characteristics									
Gender	Female	1.00							
	Male	1.28***	(1.06–1.54)						
Age group	18–29	1.00		1.00		1.00		1.00	
	30–39	0.70*	(0.47–1.05)	0.56*	(0.30–1.07)	0.69	(0.36–1.31)	0.54***	(0.35–0.82)
	40–49	0.48***	(0.33–1.70)	0.31***	(0.17–0.57)	0.40***	(0.22–0.72)	0.33***	(0.22–0.50)
	50–59	0.25***	(0.17–0.37)	0.17***	(0.09–0.31)	0.22***	(0.12–0.38)	0.27***	(0.18–0.41)
	60–69	0.15***	(0.10–0.22)	0.13***	(0.07–0.24)	0.20***	(0.11–0.36)	0.19***	(0.12–0.29)
	70+	0.10***	(0.07–0.16)	0.10***	(.0.05–0.18)	0.17***	(0.09–0.32)	0.16***	(0.10–0.26)
Marital status	Divorced, separated, and widowed			1.00					
	Married			0.61***	(0.44–0.85)				
	Single			0.56	(0.27–1.17)				
Residence	Rural	1.00		1.00					
	Urban	0.95	(0.72–1.26)	1.40**	(1.04–1.87)				
	Suburban	1.50***	(1.12–2.00)	0.92	(0.71–1.21				
Socio-economic status									
Education degree	Primary or no education	1.00						1.00	
	Secondary education	1.25*	(0.99–1.58)					1.40***	(1.12–1.74)
	College or higher	0.87	(0.63–1.18)					1.18	(0.77–1.80)
Work status	Not working	1.00		1.00		1.00		1.00	
	Non-farm work	1.66***	(1.31–2.10)	2.04***	(1.55–2.68)	2.32***	(1.61–3.32)	2.24***	(1.70–2.95)
	Farm work	1.61**	(1.12–2.31)	1.65***	(1.26–2.17)	1.75***	(1.22–2.52)	1.38**	(1.07–1.78)
Household Income (RMB/dollars)	≤¥9,999 (or ≤ $ 1.640)			1.00				1.00	
	¥10,000–49,999 (or $1,640–8,201)			1.45**	(1.06–1.98)			1.24	(0.92–1.66)
	¥50,000–99,999 (or $8,201–16,402)			1.53**	(1.06–2.21)			1.46**	(1.01–2.11)
	≥¥100,000 (or ≥ $16,402)			1.30	(0.78–2.16)			1.08	(0.65–1.79)
Self-rated social calss	Low (score 1–3)	1.00				1.00		1.00	
	Middle (score 4–5)	1.30**	(1.04–1.62)			1.37**	(1.01–1.87)	1.26**	(1.02–1.56)
	High (score 6–10)	1.46**	(1.03–2.07)			1.25	(0.67–2.33)	1.45	(0.93–2.25)
Socio-economic status compared with peers	Lower	1.00		1.00		1.00		1.00	
	Higher	1.95***	(1.25–3.03)	1.89**	(1.11–3.22)	2.25**	(1.01–4.60)	1.66*	(0.94–2.93)
	Similar	1.26**	(1.02–1.54)	1.38***	(1.10–1.72)	1.48**	(1.09–2.00)	1.27**	(1.02–1.57)
	Hard to say	1.34	(0.86–2.08)	1.23	(0.68–2.23)	0.97	(0.40–2.36)	0.93	(0.56–1.55)
Lifestyle factors									
BMI	Normal weight	1.00		1.00		1.00		1.00	
	Underweight	0.49***	(0.33–0.74)	0.75*	(0.54–1.04)	0.83	(0.46–1.50)	0.67***	(0.50–0.90)
	Over weight	0.79**	(0.65–0.96)	0.84	(0.64–1.10)	1.25	(0.89–1.75)	0.95	(0.72–1.24)
	Obese	0.38***	(0.23–0.64)	1.38	(0.62–3.07)	0.38***	(0.19–0.76)	1.06	(0.52–2.18)
Physical activity	Inactive	1.00		1.00					
	Active	1.20*	(0.99–1.45)	1.29*	(0.98–1.69)				
Psychosocial factors									
Life satisfaction	Happy life	1.00		1.00				1.00	
	Unhappy life	0.70*	(0.49–1.01)	0.64**	(0.45–0.91)			0.71**	(0.52–0.97)
	Average	0.87	(0.70–1.09)	0.74**	(0.58–0.96)			0.52***	(0.40–0.69)
Depression	Severe	1.00		1.00		1.00		1.00	
	Mild	2.48***	(1.67–3.70)	1.73***	(1.14–2.61)	1.53	(0.84–2.80)	1.81***	(1.27–2.57)
	Few or no	11.04***	(7.61–16.02)	7.59***	(5.15–11.19)	8.37***	(4.79–14.62)	6.59***	(4.73–9.16)
Relations with families and friends	Good	1.00		1.00				1.00	
	Poor	0.57***	(0.43–0.74)	0.78	(0.57–1.06)			0.82	(0.61–1.08)
	Normal	0.89	(0.73–1.07)	0.80**	(0.64–0.99)			0.66***	(0.53–0.83)
Pseudo R^2^		0.2354		0.2392		0.2274		0.2250	

Multivariate model including all significant variables through backwards stepwise logistic analysis

*OR* odds ratio, *95% CI* 95% Confidence Intervals

****p* < 0.01, ***p* < 0.05, **p* < 0.1

**Table 8:** Descriptive statistic of the total study population (*N* = 3,337)

Variables	Category	Number	Percent
Dependent variable			
Self-rated health	Good SRH	1,936	58.02
	Poor SRH	1,401	41.98
Independent variables			
Demographic characteristics			
Gender	Female	1,694	50.76
	Male	1,643	49.24
Age group	18–29	394	11.81
	30–39	615	18.43
	40–49	870	26.07
	50–59	649	19.45
	60–69	477	14.29
	70+	332	9.95
Ethnicity	Other ethnic minorities	293	8.78
	Han nationality	3,044	91.22
Marital status	Divorced,separated,and widowed	354	10.61
	Married	2,699	8.51
	Single	284	80.88
Residence	Rural	1,387	41.56
	Urban	1,950	58.44
Regions	West	878	26.31
	East	1,169	35.03
	Middle	878	26.31
	Northeast	412	12.35
Socio-economic status			
Education degree	Primary or no education	1,237	37.07
	Secondary education	1,630	48.85
	College or higher	470	14.08
Work status	Not working	1,136	34.04
	Non-farm work	1,299	38.93
	Farm work	902	27.03
Household Income (RMB/dollars)	≤¥9999 (≤$1,510)	538	16.12
	¥10000–49999($1,510–7,550)	2,026	60.71
	¥50000–99999($7,550–15,099)	499	14.95
	≥¥100000(≥$15,099)	274	8.21
Lifestyle factors			
BMI	Normal weight	2,218	66.47
	Underweight	348	10.43
	Over weight	643	19.27
	Obese	88	2.64
Physical activity	Inactive	2,109	63.20
	Active	1,228	36.80
Smoking habit	No	2,295	68.77
	Yes	1,042	31.23
Psychosocial factors			
Life satisfaction	Happy life	2,514	75.34
	Unhappy life	330	9.89
	Average	643	19.27
Depression	Severe	402	12.05
	Mild	805	24.12
	Few or no	2,130	63.83

## Appendix 2

### Robust test

**Table 9:** The distribution of SRH across socio-demographic factors, socio-economic status, lifestyle factors and psychosocial factors (*N* = 3,337)

Variables	Category	Good SRH		*X* ^2^	P
		N	%		
Demographic characteristics					
Gender	Female	925	47.78	16.44	<0.0001
	Male	1,011	52.22		
Age group	18–29	330	17.05	323.59	<0.0001
	30–39	443	22.88		
	40–49	542	28.00		
	50–59	312	16.12		
	60–69	195	10.07		
	70+	114	5.89		
Ethnicity	Other ethnic minorities	177	9.14	0.76	0.385
	Han nationality	1,759	90.86		
Marital status	Divorced, separated, and widowed	1,583	7.33	90.34	<0.0001
	Married	211	81.77		
	Single	142	10.90		
Residence	Rural	754	38.95	13.01	<0.0001
	Urban	1,182	61.05		
Regions	West	458	23.66	22.99	<0.0001
	East	707	36.52		
	Middle	487	25.15		
	Northeast	284	14.67		
Socio-economic status					
Education degree	Primary or no education	564	29.13	174.42	<0.0001
	Secondary education	1,026	53.00		
	College or higher	346	17.87		
Work status	Not working	522	26.96	206.57	<0.0001
	Non-farm work	928	47.93		
	Farm work	486	25.10		
Household Income (RMB/dollars)	≤¥9999 (≤$1,510)	214	11.05	108.39	<0.0001
	¥10000–49999($1,510–7,550)	1,196	61.78		
	¥50000–99999($7,550–15,099)	330	17.05		
	≥¥100000(≥$15,099)	196	10.12		
Lifestyle factors					
BMI	Normal weight	1,321	68.23	19.93	<0.0001
	Underweight	163	8.42		
	Over weight	381	19.68		
	Obese	45	2.32		
Physical activity	Inactive	1,163	60.07	19.40	<0.0001
	Active	773	39.93		
Smoking habit	No	1,288	66.53	10.83	0.001
	Yes	648	33.47		
Psychosocial factors					
Life satisfaction	Happy life	1,480	76.45	151.87	<0.0001
	Unhappy life	110	5.68		
	Average	313	16.17		
Depression	Severe	89	4.60	561.89	<0.0001
	Mild	298	15.39		
	Few or no	1,549	80.01

**Table 10:** Determinants of health in China: odds ratios for good self-rated health (SRH)

Variables	Category	Model 1b: Univariate analysis		Model 2b: Multivariate analysis	
		OR	95% CI	OR	95% CI
Demographic characteristics					
Gender	Female	1.00		1.00	
	Male	1.33***	(1.16–1.53)	1.29***	(1.17–1.43)
Age group	18–29	1.00		1.00	
	30–39	0.50***	(0.36–0.69)	0.48***	(0.34–0.68)
	40–49	0.32***	(0.24–0.43)	0.32***	(0.23–0.45)
	50–59	0.18***	(0.13–0.24)	0.20***	(0.14–0.28)
	60–69	0.13***	(0.10–0.19)	0.14***	(0.10–0.21)
	70+	0.10***	(0.07–0.14)	0.12***	(0.09–0.18)
Ethnicity	Other ethnic minorities	1.00			
	Han nationality	0.90	(0.70–1.15)		
Marital status	Divorced, separated, or widowed	1.00		1.00	
	Married	2.12***	(1.69–2.65)	1.36*	(1.06–1.73)
	Single	4.32***	(3.07–6.07)	0.97	(0.63–1.48)
Residence	Rural	1.00			
	Urban	1.29***	(1.12–1.49)		
Regions	West	1.00		1.00	
	East	1.40***	(1.18–1.67)	1.18	(0.95–1.48)
	Middle	1.14	(0.95–1.38)	1.14	(0.91–1.42)
	Northeast	2.03***	(1.59–2.61)	1.69***	(1.26–2.26)
Socio-economic status					
Education degree	Primary or no education	1.00			
	Secondary education	2.03***	(1.73–2.36)		
	College or higher	3.33***	(2.64–4.61)		
Work status	Not working	1.00		1.00	
	Non-farm work	2.94***	(2.49–3.48)	1.63***	(1.31–2.03)
	Farm work	1.37***	(1.15–1.64)	1.48***	(1.19–1.86)
Household Income (RMB/dollars)	≤¥9999 (≤$1,510)	1.00		1.00	
	¥10000–49999 ($1,510–7,550)	2.18***	(1.80–2.65)	1.25*	(0.99–1.59)
	¥50000–99999 ($7,550–15,099)	2.96***	(2.30–3.81)	1.17	(0.86–1.61)
	≥¥100000 (≥$15,099)	3.80***	(2.78–5.21)	1.34	(0.91–1.97)
Lifestyle factors					
BMI	Normal weight	1.00		1.00	
	Underweight	0.60***	(0.48–0.75)	0.65***	(0.49–0.85)
	Over weight	0.98	(0.82–1.18)	0.96	(0.78–1.18)
	Obese	0.71	(0.46–1.08)	0.60**	(0.39–0.98)
Physical activity	Inactive	1.00		1.00	
	Active	1.38***	(1.20–1.60)	1.15*	(0.95–1.37)
Smoking habit	No	1.00			
	Yes	1.29***	(1.11–1.49)		
Psychosocial factors					
Life satisfaction	Happy life	1.00		1.00	
	Unhappy life	0.28***	(0.22–0.36)	0.55***	90.41–0.74)
	Average	0.53***	(0.45–0.64)	0.67***	(0.54–0.82)
Depression	Severe	1.00		1.00	
	Mild	2.07***	(1.57–2.72)	1.84***	(1.37–2.48)
	Few or no	9.38***	(7.27–12.09)	7.45***	(5.63–9.84)
Pseudo R^2^				0.21	

Multivariate model including all significant variables through backwards stepwise logistic analysis

*OR* odds ratio, *95% CI* 95% Confidence Intervals

****p* < 0.01, ***p* < 0.05, **p* < 0.1

**Table 11:** Examine the extent to which lifestyle or psychosocial factors explained the effect of socio-economic status on good SRH

Variables	Category	Model 3b		Model 4b		Model 5b	
		OR	95% CI	OR	95% CI	OR	95% CI
Demographic characteristics							
Gender	Female	1.00		1.00		1.00	
	Male	1.22***	(1.03–1.45)	1.18*	(0.97–1.43)	1.22**	(1.02–1.45)
Age group	18–29	1.00		1.00		1.00	
	30–39	0.41***	(0.28–0.60)	0.39***	(0.26–0.57)	0.45***	(0.30–0.67)
	40–49	0.27***	(0.19–0.40)	0.25***	(0.17–0.37)	0.30***	(0.20–0.45)
	50–59	0.17***	(0.11–0.25)	0.15***	(0.10–0.22)	0.19***	(0.12–0.28)
	60–69	0.15***	(0.10–0.22)	0.13***	(0.09–0.20)	0.13***	0.08–0.20)
	70+	0.13***	(0.08–0.20)	0.12***	(0.08–0.19)	0.11***	(0.07–0.18)
Marital status	Divorced, separated, and widowed	1.00		1.00		1.00	
	Married	1.15	(0.89–1.48)	1.14	(0.88–1.47)	0.94	(0.71–1.25)
	Single	0.74	(0.47–1.16)	0.73	(0.47–1.15)	0.71	(0.44–1.15)
Residence	Rural	1.00		1.00		1.00	
	Urban	1.03	(0.84–1.26)	0.98	(0.80–1.20)	1.03	(0.83–1.29)
Regions	West	1.00		1.00		1.00	
	East	1.40***	(1.14–1.72)	1.37***	(1.11–1.69)	1.20	(0.96–1.50)
	Middle	1.23**	(1.00–1.51)	1.23**	(1.00–1.51)	1.14	(0.92–1.42)
	Northeast	2.02***	(1.55–2.63)	2.04***	(1.56–2.67)	1.69***	(1.26–2.26)
Socio-economic status							
Education degree	Primary or no education	1.00		1.00		1.00	
	Secondary education	1.21**	(1.02–1.46)	1.17	(0.97–1.40)	1.09	(0.89–1.32)
	College or higher	1.32*	(0.98–1.77)	1.23	(0.90–1.66)	1.16	(0.85–1.60)
Work status	Not working	1.00		1.00		1.00	
	Non-farm work	1.61***	(1.31–1.98)	1.61***	(1.30–1.98)	1.58***	(1.26–1.97)
	Farm work	1.51***	(1.19–1.91)	1.49***	(1.18–1.89)	1.50***	(1.16–1.94)
Household Income (RMB/dollars)	≤¥9999 (≤$1,510)	1.00		1.00		1.00	
	¥10000–49999 ($1,510–7,550)	1.54***	(1.25–1.92)	1.52***	(1.22–1.88)	1.25*	(0.98–1.59)
	¥50000–99999 ($7,550–15,099)	1.77***	(1.31–2.37)	1.68***	(1.25–2.27)	1.17	(0.85–1.62)
	≥¥100000 (≥$15,099)	1.94***	(1.34–2.79)	1.85***	(1.28–2.68)	1.34	(0.90–1.99)
Lifestyle factors							
BMI	Normal weight			1.00			
	Underweight			0.65***	(0.50–0.84)		
	Over weight			1.01	(0.83–1.23)		
	Obese			0.70	(0.44–1.12)		
Physical activity	Inactive			1.00			
	Active			1.30***	(1.09–1.53)		
Smoking habit	No			1.00			
	Yes			1.18	(0.96–1.44)		
Psychosocial factors							
Life satisfaction	Happy life					1.00	
	Unhappy life					0.56***	(0.42–0.75)
	Average					0.68***	(0.55–0.83)
Depression	Severe					1.00	
	Mild					1.87***	(1.39–2.51)
	Few or no					7.55***	(5.72–9.96)
Pseudo R^2^		0.1154		0.1806		0.2071	

Model 3b controlled for demographic characteristics (except ethnicity variable, which was not correlated with SRH in univariate analysis) and socio-economic status factors

Model 4b also controlled lifestyle factors in addition to the factors controlled in model 3b

Model 5b also controlled for psychosocial factors in addition to the factors controlled in model 3b

*OR* odds ratio, *95% CI* 95% Confidence Intervals

****p* < 0.01, ***p* < 0.05, **p* < 0.1

Note:

Data were from the Chinese General Social Survey (2010). The structure of the questionnaire in 2010 is similar to that in 2013

Since only 3,337 respondents reported smoking habit, so the data from these 3,337 respondents were complete

“Two subjective socio-economic status” variables and “Relationship with families and friends” can not be obtained from the dataset 2010. But we add “Smoking habit” variable (Yes = 1 No = 0) in the analysis

## Abbreviations

BMI: Body mass index
CGSS: Chinese General Social Survey
CIs: Confidence intervals
ORs: Odds ratios
SRH: Self-rated health

## Footnotes

1 There were 31 provinces, municipalities, and autonomous regions in mainland China. This survey covered 28 provinces, municipalities, and autonomous regions except Xinjiang, Tibet and Hainan.

2 Eastern region included 9 provinces: Beijing, Tianjin, Hebei, Shandong, Jiangsu, Shanghai, Zhejiang, Fujian, Guangdong.

3 Middle region included 6 provinces: Shanxi, Henan, Hubei, Hunan, Jiangxi and Anhui.

4 Northeast region included 3 provinces: Heilongjiang, Jilin and Liaoning.

5 Western region included 10 provinces: Chongqing, Sichuan, Guangxi, Guizhou, Yunnan, Shanxi, Gansu, Neimenggu, Ningxia and Qinghai.
